# First Insights into the Genome Sequence of the Cellulolytic Bacterium *Clostridium hungatei* DSM 14427

**DOI:** 10.1128/genomeA.00363-17

**Published:** 2017-05-18

**Authors:** Anja Poehlein, Katrina Funkner, Miriam A. Schüler, Rolf Daniel

**Affiliations:** a Genomic and Applied Microbiology & Göttingen Genomics Laboratory, Institute of Microbiology and Genetics, Georg-August University of Göttingen, Göttingen, Germany; b Members of the Applied Bioinformatics in Microbiology Course of the Microbiology and Biochemistry M.Sc./Ph.D. program, Georg-August University of Göttingen, Göttingen, Germany

## Abstract

*Clostridium hungatei* is an obligate anaerobic and spore-forming bacterium, which was isolated from soil. It ferments carbohydrates, such as cellulose or d-glucose. *C. hungatei* is able to fix nitrogen. The draft genome consists of 1 chromosome (4.902 Mb) with 4,246 predicted protein-coding genes.

## GENOME ANNOUNCEMENT

*Clostridium hungatei* DSM 14427 is an obligate anaerobic, spore-forming, and mesophilic bacterium. It is able to fix nitrogen and is motile (1). *C. hungatei* was isolated from soil under a pile of rotting wood chips (1). Gram staining showed a Gram-negative reaction, but the cell wall exhibited an untypical multilayer structure (1). Comparative 16S rRNA gene analysis classified *C. hungatei* together with other cellulolytic organisms, such as *Clostridium cellulolyticum* and *Clostridium cellobioparum*, as a member of cluster III of clostridia (1).

Chromosomal DNA isolation of *C. hungatei* DSM 14427 was performed using the MasterPure complete DNA purification kit, as described by the supplier (Epicentre, Madison, WI, USA). The DNA extract was used as recommended by the manufacturer (Illumina, San Diego, CA, USA) to generate Illumina paired-end sequencing libraries. Libraries were sequenced using a MiSeq instrument and MiSeq reagent kit version 3, according to the protocol of the manufacturer (Illumina). Quality filtering of the recovered reads was performed using Trimmomatic version 0.36 (2) and resulted in 2,612,420 paired-end reads. The assembly was performed using SPAdes genome assembler software version 3.10.0 (3) Assembly yielded 102 contigs (>500 bp), with an average coverage of 119-fold. Qualimap version 2.1 was employed for assembly validation (4). The draft genome size was 4.902 Mb, with a G+C content of 42.27%. Automatic gene prediction was performed using the software tool Prokka (5) and resulted in 3,010 protein-coding genes with a predicted function and 1,236 genes encoding hypothetical proteins. Additionally, genes encoding 57 tRNAs, 9 rRNAs, and 1 transfer-messenger RNA (tmRNA) were identified. Interestingly, 4 predicted protein-coding genes were associated with a clustered regularly interspaced short palindromic repeat (CRISPR)-associated (Cas) system that belonged to subtype I-C/DVULG, according to the classification of Makarova et al. (6). In line with the presence of CRISPR-Cas genes, 20 putative phage-associated genes were identified, including a putative integrase of the phage phiRv2.

The fermentation products of *C. hungatei* from various carbohydrates, such as cellobiose, cellotriose, cellotetraose, cellopentaose, d-glucose, d-fructose, d-xylose, xylan, and gentiobiose, are acetate, ethanol, H_2_, and CO_2_ (1). Correspondingly, putative genes for the utilization of cellobiose, cellulose, glucose, fructose, mannose, xylase, and xylan were present in the genome of *C. hungatei*. Although *C. hungatei* is closely related to *C. cellulolyticum*, the compositions of the cellulase system of these two species are strikingly different (1). *C. hungatei* possesses genes encoding nitrogenase clusters (*anf-nif-vnf*) for nitrogen fixation, which correspond to the clusters present in *Clostridium pasteurianum* (7). Furthermore, 38 putative genes coding for antibiotic resistances were present, of which 14 genes code for multidrug resistances. Additionally, 11 potential multidrug export systems were annotated. In contrast to previous experiments (1), putative genes encoding tetracycline and chloramphenicol resistance were found to be present in the genome sequence of *C. hungatei*.

### Accession number(s).

The whole-genome shotgun project has been deposited at DDBJ/EMBL/GenBank under the accession number MZGX00000000. The version described here is version MZGX01000000.
